# Long-acting dolutegravir formulations prevent neurodevelopmental impairments in a mouse model

**DOI:** 10.3389/fphar.2023.1294579

**Published:** 2023-12-12

**Authors:** Emma G. Foster, Brady Sillman, Yutong Liu, Micah Summerlin, Vikas Kumar, Balasrinivasa R. Sajja, Adam R. Cassidy, Benson Edagwa, Howard E. Gendelman, Aditya N. Bade

**Affiliations:** ^1^ Department of Pharmacology and Experimental Neuroscience, University of Nebraska Medical Center, Omaha, NE, United States; ^2^ Department of Radiology, University of Nebraska Medical Center, Omaha, NE, United States; ^3^ Department of Genetics, Cell Biology, and Anatomy, University of Nebraska Medical Center, Omaha, NE, United States; ^4^ Departments of Psychiatry and Psychology & Pediatric and Adolescent Medicine, Mayo Clinic, Rochester, MN, United States; ^5^ Department of Pharmaceutical Sciences, University of Nebraska Medical Center, Omaha, NE, United States

**Keywords:** pregnancy, dolutegravir, HIV-1, long-acting nanoformulations, neurodevelopment

## Abstract

The World Health Organization has recommended dolutegravir (DTG) as a preferred first-line treatment for treatment naive and experienced people living with human immunodeficiency virus type one (PLWHIV). Based on these recommendations 15 million PLWHIV worldwide are expected to be treated with DTG regimens on or before 2025. This includes pregnant women. Current widespread use of DTG is linked to the drug’s high potency, barrier to resistance, and cost-effectiveness. Despite such benefits, potential risks of DTG-linked fetal neurodevelopmental toxicity remain a concern. To this end, novel formulation strategies are urgently needed in order to maximize DTG’s therapeutic potentials while limiting adverse events. In regard to potential maternal fetal toxicities, we hypothesized that injectable long-acting nanoformulated DTG (NDTG) could provide improved safety by reducing drug fetal exposures compared to orally administered native drug. To test this notion, we treated pregnant C3H/HeJ mice with daily oral native DTG at a human equivalent dosage (5 mg/kg; n = 6) or vehicle (control; n = 8). These were compared against pregnant mice injected with intramuscular (IM) NDTG formulations given at 45 (n = 3) or 25 (n = 4) mg/kg at one or two doses, respectively. Treatment began at gestation day (GD) 0.5. Magnetic resonance imaging scanning of live dams at GD 17.5 was performed to obtain T_1_ maps of the embryo brain to assess T_1_ relaxation times of drug-induced oxidative stress. Significantly lower T_1_ values were noted in daily oral native DTG-treated mice, whereas comparative T_1_ values were noted between control and NDTG-treated mice. This data reflected prevention of DTG-induced oxidative stress when delivered as NDTG. Proteomic profiling of embryo brain tissues harvested at GD 17.5 demonstrated reductions in oxidative stress, mitochondrial impairments, and amelioration of impaired neurogenesis and synaptogenesis in NDTG-treated mice. Pharmacokinetic (PK) tests determined that both daily oral native DTG and parenteral NDTG achieved clinically equivalent therapeutic plasma DTG levels in dams (4,000–6,500 ng/mL). Importantly, NDTG led to five-fold lower DTG concentrations in embryo brain tissues compared to daily oral administration. Altogether, our preliminary work suggests that long-acting drug delivery can limit DTG-linked neurodevelopmental deficits.

## Introduction

The World Health Organization (WHO) and the Department of Health and Human Services (DHHS) recommend antiretroviral therapy (ART) for pregnant women living with human immunodeficiency virus type-1 (HIV-1) (World Health Organization WHO, 2016; U.S. Department of Health and Human Services, 2023). ART serves to improve maternal health and reduce vertical, mother to fetus, HIV-1 transmission (World Health Organization WHO, 2019a; U.S. Department of Health and Human Services, 2023). Notably, ART has significantly reduced viral transmission to less than 1% from infected mothers to the fetus during gestation (The Centers for Disease Control and Prevention (CDC); Peters et al., 2017; Schnoll et al., 2019; Rasi et al., 2022). This is noteworthy as more than 15 million women of child-bearing age are living with HIV-1 infection (LWHIV), worldwide (The Joint United Nations Programme on HIV/AIDS UNAIDS, 2023), and up to 1.3 million pregnant women LWHIV give birth each year (Ramokolo et al., 2019; Crowell et al., 2020). Due to the growing availability of antiretroviral drugs (ARVs), including in resource-limited countries (RLCs), more than 80% of pregnant women LWHIV now have access to ART (The Joint United Nations Programme on HIV/AIDS UNAIDS, 2023). However, along with the significant benefits of ART in preventing viral transmission and associated morbidities, risks of adverse events in fetuses linked to gestational ARVs exposure remain (Hill et al., 2018). With over a million ART-exposed HIV-1 uninfected (AEHU) children born each year (Ramokolo et al., 2019; Crowell et al., 2020), a recognition of ARV-associated adverse events during pregnancy remains timely.

Dolutegravir (DTG) is currently an integral part of antiretroviral regimens (ART) for treatment of people LWHIV (World Health Organization WHO, 2016; Department of Health and Human Services DHHS, 2023). This is due to high drug potency, limited drug-drug interactions, and high genetic barrier to resistance (Bre et al., 2017; Dorward et al., 2018; Hill et al., 2018). These also underscore why DTG is prescribed in both resource-rich and -limited countries (RRCs and RLCs). Moreover, a pricing agreement was made in 2017 to accelerate the availability of generic DTG-based regimen in RLCs at the cost of $75 US dollars/person/year. As a result, up to 100 RLCs have transitioned to DTG-based regimen by mid-2020 (The Lancet, 2020). Thus, nearly seven million people had access to this DTG-based regimen in 2019, and up to 15 million people are expected to be treated with it by 2025 (Dorward et al., 2018; Hill et al., 2018; World Health Organization WHO, 2018; The Lancet, 2020). This includes women of child-bearing age (The Joint United Nations Programme on HIV/AIDS UNAIDS, 2023). In 2022, 3,100 adolescent girls and young women aged 15–24 years became HIV-1 infected each week in sub-Saharan Africa (The Joint United Nations Programme on HIV/AIDS UNAIDS, 2023). Moreover, increasing pretreatment resistance (PDR) to non-nucleoside reverse transcriptase inhibitors (NNRTIs) in RLCs has led to increased usage preference for DTG-based regimens (World Health Organization WHO, 2019a; World Health Organization WHO, 2019b). Due to the current inclusion of DTG-based regimens in guidelines for the treatment of pregnant women LWHIV or those of child-bearing age (World Health Organization WHO, 2019a; U.S. Department of Health and Human Services, 2023) and rising pretreatment resistance to NNRTIs in RLCs, a majority of women LWHIV are or will be treated with DTG.

Concerns for DTG usage by pregnant women or women of child-bearing age LWHIV remain. These were first noted by a recorded risk of fetal congenital abnormalities in 2018 (Zash et al., 2018a; Zash et al., 2019; Crowell et al., 2020). The interim analysis of the Tsepamo birth surveillance study in Botswana reported neural tube defects (NTDs) in babies born to mothers who received DTG periconceptionally. The numbers for DTG-linked NTDs recorded were eight times higher than in those born to mothers who had taken other ARVs at conception (Zash et al., 2018a). Continued surveillance in Botswana showed a lower risk of NTDs but remained 2–3 times higher than other ARVs at conception (Zash et al., 2019). These numbers have continued to decrease as the Tsepamo study, most recently, reported a similar rate of birth defects between DTG and other ARVs usage at the time of conception in a late breaking abstract at the 24th International AIDS Conference, 2022. Nonetheless, the assessment of fetal congenital defects in Botswana remains operative. Notably, DTG-associated fetal defects were reported in experimental systems including studies of pregnant mice who were given the drug at therapeutic levels (Mohan et al., 2020; Tukeman et al., 2023). Furthermore, the Surveillance Monitoring for ART Toxicities (SMARTT) study, an observational study in North America, reported associations between *in utero* DTG exposures and neurologic abnormalities in children during postnatal development (Crowell et al., 2020). Similarly, our research demonstrated that DTG-induced inhibition of matrix metalloproteinases (MMPs) in mice fetal brains during gestation can affect postnatal neurodevelopment with noted neuroinflammation and neuronal injury in adolescent pups. Altogether, both clinical and pre-clinical studies noted the risk of neurodevelopmental toxicities following *in utero* DTG exposure. However, due to higher benefit-to-risk ratio, DTG is currently recommended during pregnancy irrespective of trimester. Special mentions included fewer mother-to-child HIV-1 transmission and maternal deaths, and cost-effective regimens (Dugdale et al., 2019; Phillips et al., 2020). Altogether, with the acknowledgement that millions of fetuses are and will be exposed to DTG *in utero*, development of novel strategies to maximize the benefits of DTG and minimize its adverse events during pregnancy would serve to improve its safety and therapeutic benefits in pregnancy.

With emerging clinical usage of long-acting ARV nanoformulations for treatment and prevention therapies [LA-cabotegravir (CAB); integrase strand transfer inhibitor (INSTI)] (U S Food and Drug Administration FDA, 2021a; U S Food and Drug Administration FDA, 2021b), transformation of the more potent and broadly used DTG and potentially other ARVs into LA formulations would elicit improved safety profiles during pregnancy. Based on such notion, LA DTG formulations (NDTG) were tested in pregnant mice. The data demonstrated that NDTG achieve sustained therapeutic DTG levels in the mother while lowering drug exposure to the embryo brain. This data was further associated with reductions of DTG-linked oxidative stress and with attenuation of neuronal and synaptic impairments in the fetal brain. To this end, we show that LA parenteral formulations could minimize embryo brain DTG exposures and thus, potentially limit drug-related neurodevelopmental toxicities.

## Materials and methods

### Nanoparticle preparation and characterization

NDTG was prepared in endotoxin-free water, pH 7.0 at a ratio of 10:1 (w/w) drug:poloxomer 407 (P407) and starting drug concentration of 5% (w/v). P407 was purchased from Sigma-Aldrich (St. Louis, MO, United States). HyPure endotoxin-free cell culture grade water was purchased from Cytiva (Marlborough, MA, United States). The pre-suspension was homogenized on an Avestin EmulsiFlex-C3 high-pressure homogenizer (Ottawa, ON, Canada) at 20,000 ± 1,000 PSI with the chiller set to 6°C to form the desired particle size. Nanoparticles (100-fold dilution in water) were characterized for hydrodynamic particle diameter (size), polydispersity index (PDI), and zeta potential as measured by dynamic light scattering (DLS) using a Malvern Zetasizer Nano-ZS (Worcestershire, UK). For quality control, selection criteria were set to nanoformulations with size 200–500 nm, PDI at 0.25 ± 0.05, and encapsulation efficiency greater than 75%. Nanoformulations with these selection criteria have achieved stabilization of particles and extended pharmacokinetics (PK) profile (Sillman et al., 2018; Kulkarni et al., 2020; Deodhar et al., 2022). Scanning electron microscopy (SEM) was performed to evaluate structural morphology of nanoparticles (Sillman et al., 2018). All formulations were suitably syringable and non-viscous enough to pass through a 28 G needle. Further, these samples were extracted in methanol and were analyzed using UPLC-TUV for the drug content. Drug was extracted from the nanoformulation in HPLC-grade methanol (1,000-fold dilutions). A Waters ACQUITY UPLC H-Class system with tunable ultraviolet/visible (TUV) detector and Empower 3 software were used to measure drug concentrations of nanoformulation. A Phenomenex Kinetex 5 μm C18 column (150 × 4.6 mm) (Torrance, CA, United States) was used to separate DTG. Further, DTG was detected at 254 nm, using isocratic elution with a mobile phase consisting of 65% 50 mM potassium phosphate monobasic (KH_2_PO_4_), pH 3.2/35% HPLC-grade Acetonitrile (ACN) at a flow rate of 1.0 mL/min. HPLC-grade methanol, KH_2_PO_4_, and HPLC-grade ACN were purchased from Fisher Scientific (Waltham, MA, United States). Drug content was determined relative to peak areas of drug standards (0.05–50 μg/mL) in methanol. Encapsulation efficiency (EE%) was calculated using the following formula: Encapsulation efficiency (EE%) = (Drug_encapsulated in formulation_ [mg]/Drug_total added to premix_ [mg]) x 100%.

### Study approvals

Animal studies were approved by the University of Nebraska Medical Center (UNMC) Institutional Animal Care and Use Committee (IACUC) in accordance with the standards of the Guide for the Care and Use of Laboratory Animals (National Research Council of the National Academies, 2011).

### Animals

C3H/HeJ mice (male and female, 10–12 weeks of age), were utilized for all animal experiments. Mice were purchased from the Jackson Laboratory (Bar Harbor, ME) and were housed in microisolator cages in climate‐controlled laboratory animal facilities at UNMC. Mice were maintained on a 12‐hour light‐dark cycle and were given free access to irradiated rodent feed (TD. 180911, Envigo Teklad diet, Madison, WI) and sterile water. Animals were acclimated to the animal facility environment for approximately 1–2 weeks prior to experiments. Healthy animals were selected by observing behavior, movement, and weight. For timed mating, a female was placed with a male overnight. The following morning the male was removed from the cage and the female was evaluated for a positive vaginal plug. Females presenting vaginal plugs were weighed and randomly distributed to treatment (DTG or NDTG) or control (vehicle) group. Male mice were utilized only for mating and did not receive any drug/chemical. The time of conception was considered at midnight on the evening of the mating. Thus, treatment of plugged females was started the day of the positive vaginal plug detection and this day was identified as gestation day (GD) 0.5.

### Study groups

Pregnant mice (C3H/HeJ) identified by positive vaginal plugs were randomly distributed in four treatment groups. In group one, pregnant female mice (dams) were treated orally every day with native DTG at human therapeutic equivalent dosage (5 mg/kg, mouse weight equivalent; n = 6) by oral gavage starting at gestation day (GD) 0.5 up to GD 16.5. In group two, dams were treated orally every day with the vehicle (n = 8) used to prepare DTG solution for oral administration [Control; dimethylsulfoxide:Solutol^®^:50 mM *N*-methylglucamine in 3% mannitol (1:1:8, v:w:v)] by oral gavage. Volume and administration scheme for vehicle was similar to native DTG oral administration. In group three, dams were injected with two NDTG (25 mg/kg, mouse weight equivalent; n = 4) intramuscular (IM) injections, first at GD 0.5 and second at GD 9.5. In group four, dams were injected with a single IM injection of NDTG (45 mg/kg, mouse weight equivalent; n = 3) at GD 0.5. Effect of oral native DTG administration or intramuscular (IM) injectable NDTG exposure during pregnancy on mice embryo development was evaluated in all four study groups (Group 1–4). Weight gain in dams was measured on GD 0.5, 8.5 and 15.5. Dams were humanely euthanized, and embryos and placentas were harvested at GD 17.5. Embryos were evaluated for NTDs. At the time of harvest, the total number of implants (litter size, resorption rates or viable embryos) were recorded for each pregnancy. Further, whole brain tissues from normal viable embryos were isolated and processed for tissue DTG concentrations and global proteomic evaluations.

### *In utero* magnetic resonance imaging (MRI)

*In utero* magnetic resonance imaging (MRI) was performed on mice embryo brain for T_1_ mapping on a 7 T/16 cm Bruker PharmaScan or a 7 T 21 cm Bruker Biospec (Karlsure, Germany) MRI/MRS system. At GD 17.5, live dams from all four study groups (Group 1–4) were scanned to compute T_1_ maps to examine T_1_-relaxivity of oxidative stress in embryo brain. Mice were anesthetized using 1.5%–2.5% isoflurane carried by oxygen at the flow rate of 1 L/minute. Breathing rate and body temperature of dams were observed during MRI to ensure the health. T_2_-weighted images were first acquired in both axial and coronal directions to locate a fetal brain for T_1_ mapping. Under the guidance of the T_2_-weighted images, imaging slices for T_1_ mapping were positioned in the coronal direction of the embryo brain, i.e., the anterior-posterior direction. For T_1_ mapping, a Rapid Acquisition with Refocused Echoes (RARE) sequence with 8 TRs = 120, 250, 350, 500, 1,000, 1,500, 3,000, 7,500 ms, TE = 32 ms, 5 slices with slice thickness = 0.5 mm, FOV = 35 × 25 mm^2^, matrix = 192 × 192 images were acquired. T_1_ relaxation times maps were created by fitting the RARE data with multiple TR values to the corresponding mathematical model of the MR signal at each pixel. The data fitting was performed using the Levenberg-Marquardt method. Custom computer codes were written in Interactive Data Language (IDL 8.8; L3Harris Geospatial Solutions, Inc. Broomfield, Colorado, United States, 2022) to generate these maps. Further, for quantitative assessments, average T_1_-relaxation times were calculated on T_1_ maps by region-of-interest (ROI) analysis using the MIPAV (Medical Image Processing, Analysis, and Visualization) software. Animals from all four study groups were scanned. Two embryos per dam were randomly selected to acquire T_1_ maps on the embryo brains.

### Label free proteomics and data analysis

After MRI imaging, dams were humanely euthanized and whole embryo brains were isolated. Embryo brain tissues were flash frozen in liquid nitrogen and stored in −80°C. Later, embryo whole brain tissues were homogenized in NP-40 lysis buffer. Tissue homogenates were centrifuged at 16,000 × g at 4°C for 20 min and supernatants were collected. Total protein levels in each sample were quantitated using the Pierce™ BCA Protein Assay Kit (Thermo Fisher Scientific). Minimum five embryo brains per group were selected. Each sample was from a different dam for all groups. For non-targeted proteomic profiling, protein samples from both NDTG groups (25 and 45 mg/kg) were pooled together to strengthen the biological assessment as no differences were observed for total DTG levels or T_1_ mapping of oxidative stress between these two groups. 50 μg of protein per sample was used for proteomic analysis. Following chloroform/methanol extraction to remove detergents, the protein pellet was resuspended using 100 mM ammonium bicarbonate. Protein was then digested using MS-grade trypsin (Pierce) overnight at 37°C. Samples then underwent reduction using 10 mM DTT at 56°C for 30 min followed by alkylation with 50 mM iodoacetamide at room temperature for 25 min. Peptides were then cleaned with PepClean C18 spin columns (Thermo) and resuspended in 2% acetonitrile (ACN) and 0.1% formic acid (FA). 500 ng of each sample was then loaded onto a trap column (Acclaim PepMap 100 75 μm × 2 cm C18 LC Column; Thermo Scientific™) at a flow rate of 4 μL/min then separated with a Thermo RSLC Ultimate 3,000 (Thermo Scientific™) on a Thermo Easy-Spray PepMap RSLC C18 75 μm × 50 cm C-18 2 µm column (Thermo Scientific™) with a step gradient of 4%–25% solvent B (0.1% FA in 80% ACN) from 10 to 100 min and 25%–45% solvent B for 100–130 min at 300 nL/min and 50°C with a 155 min total run time. Eluted peptides were then analyzed by a Thermo Orbitrap Exploris 480 (Thermo Scientific™) mass spectrometer in a data dependent acquisition mode. A survey full scan MS (from m/z 350–1,200) was acquired in the Orbitrap with a resolution of 60,000. The normalized AGC target for MS1 was set as 300% and ion filling time was set as 25 ms. The highest intensity ions with charge state of 2-6 were isolated in a 3 s cycle and fragmented using HCD fragmentation with 30% normalized collision energy then detected at a mass resolution of 15,000 at 200 m/z. The AGC target for MS/MS was set as 50% and ion filling time set to auto for 30 s with a 10 ppm mass window. Protein identification was achieved by searching MS/MS data against the swiss-prot Rat protein database which was downloaded on November 2022 using the in-house PEAKS X + DB search engine. This search was set up for full tryptic peptides with a maximum of two missed cleavage sites. Variable modifications were acetylation of protein N-terminus and oxidized methionine. The fixed modification was carbamidomethylation. Precursor mass tolerance threshold was set as 10 ppm and maximum fragment mass error was 0.02 Da. The significance threshold of the ion score was calculated based on a false discovery rate of ≤1%. Quantitative data analysis was performed using progenesis QI proteomics 4.2 (Nonlinear Dynamics). Statistical analysis for proteomic data was performed using ANOVA. The Benjamini–Hochberg (BH) method was used to adjust *p* values for multiple-testing caused false discovery rate. The adjusted *p* ≤ 0.05 was considered as significant. Various plots such as volcano and PCA plots were generated using Partek Genomics Suite 7.0. Identified proteins were analyzed for biological processes and functional pathways using ingenuity pathway analysis (IPA) and ShinyGo 0.77. Heatmaps were generated using GraphPad Prism 9.0 software (La Jolla, CA).

### DTG PK and biodistribution (BD) tests

PK and BD profiles of oral native DTG administration or intramuscular (IM) injectable NDTG were determined. Blood samples were collected from dams in heparinized tubes by cheek puncture (submandibular vein) using a 5 mm lancet (MEDIpoint, Inc., Mineola, NY) at GD 16.5. Collected blood samples were centrifuged at 2,000 × g for 8 min and plasma was collected for the quantitation of plasma DTG levels. Further, biodistribution of DTG to placental tissue and embryo brain tissue was determined at GD 17.5. DTG concentrations were quantitated in dams’ plasma, and tissue (placenta or embryo brain tissue) homogenates by ultra-performance liquid chromatography tandem mass spectrometry (UPLC-MS/MS) using a Waters ACQUITY Premier UPLC (Milford, MA, United States) connected to a Xevo TQ-XS mass spectrometer and analyzed using Waters MassLynx V4.2 software (Milford, MA, United States) according to previously published protocols (Sillman et al., 2018; Bade et al., 2021; Deodhar et al., 2022). Each plasma or tissue (placenta and embryo brain) sample was randomly selected for DTG measures from distinct litter. All solvents for sample processing and UPLC-MS/MS analysis were Optima grade (Fisher).

For DTG quantitation, chromatographic separation of a 10 μL sample injection was performed on a Waters ACQUITY UPLC BEH Shield RP18 column (1.7 μm, 2.1 mm × 100 mm) at 25°C using a 10-min gradient method. Mobile phase A consisted of 7.5 mM ammonium formate in water, adjusted to pH 3 using formic acid and mobile phase B consisted of 100% ACN at a flow rate of 0.25 mL/min. For the first 5 min, the mobile phase composition was 40% B. Afterwards, a cleaning step consisting of 95% B was held constant for 3 min before equilibrating the column back to initial conditions for 2 min. Capillary voltage of 3.3 kV was used for DTG and DTG-d3 multiple reaction monitoring (MRM) transitions of 420.16 > 277.17 and 423.18 > 135.96 using collision energies of 26 eV and 54 eV and cone voltages of 6 V and 28 V, respectively. All quantitations were determined using analyte peak area to internal standard peak area ratios.

### Statistical analysis

Statistical analyses were conducted using GraphPad Prism 9.0 software (La Jolla, CA). Data were expressed as mean ± standard error of the mean (SEM) with a minimum of 3 biological replicates. For comparisons between two groups, Student’s t-test (two-tailed) was used. A one-way ANOVA followed by Tukey’s or Dunnett’s test was used to compare three or more groups. Statistical significance was denoted as ^#^ *p* < 0.1, *p < 0.05, **p < 0.01, ***p < 0.001, ****p < 0.0001.

## Results

### DTG-nanoformulations limit embryo brain drug exposures

**FIGURE 1:**
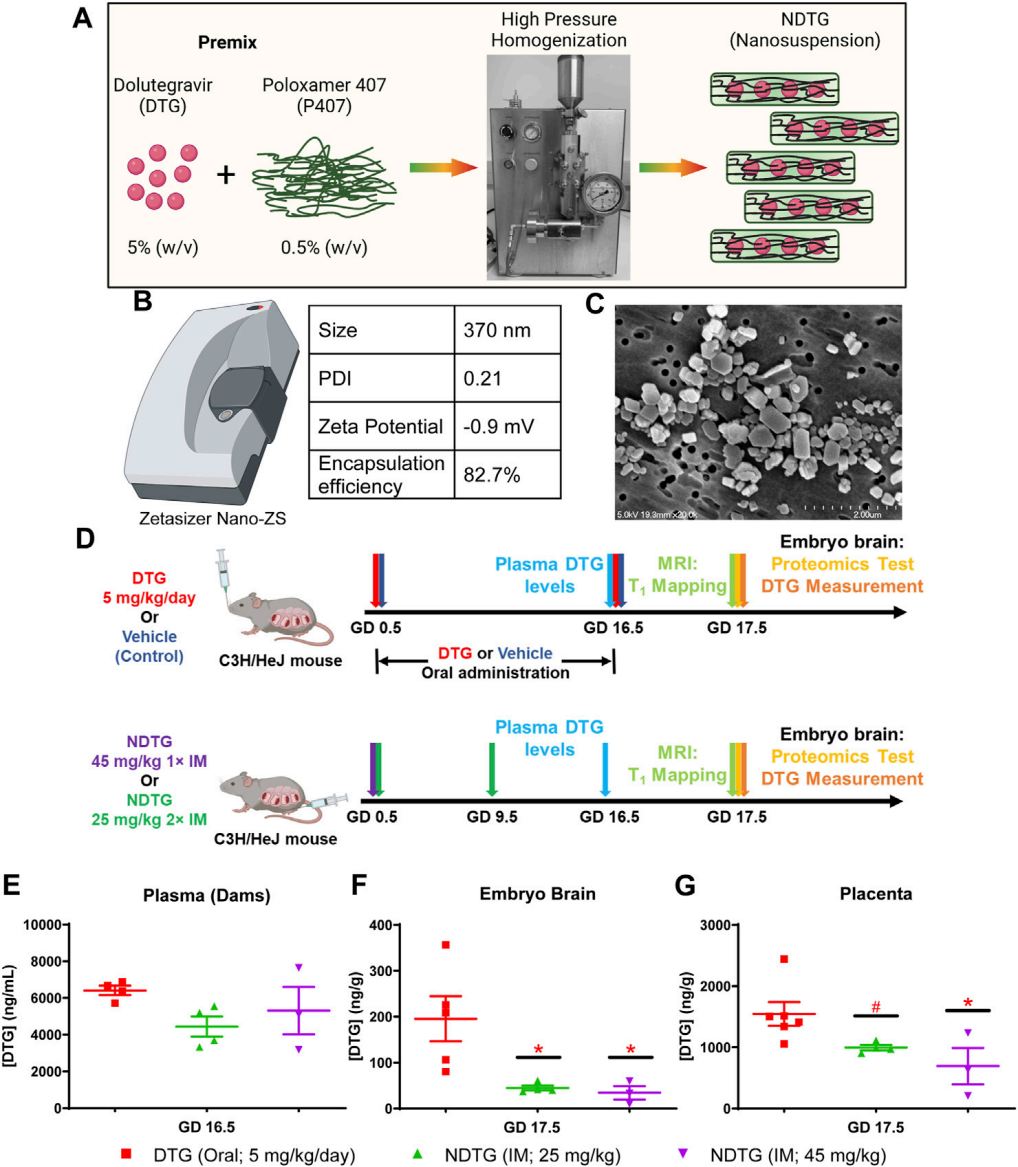
PK and BD profiles of NDTG during pregnancy. **(A)** Pre-mixture of dolutegravir (DTG) and Poloxamer 407 (P407) were subjected to homogenization using an Avestin EmulsiFlex-C3 high-pressure homogenizer to synthesize uniform NDTG particles. **(B)** NDTG particles were characterized for hydrodynamic particle diameter (size), polydispersity index (PDI), and zeta potential using a Malvern Zetasizer Nano-ZS. Drug content of formulation was measured using UPLC-TUV. **(C)** Morphological assessment of NDTG particles was performed using scanning electron microscopy (SEM). **(D)** Schematic representation of timeline and study design. Pregnant C3H/HeJ female mice (dams) were randomly distributed in four study groups. In group one, dams were daily administered native DTG at 5 mg/kg dose by oral gavage from gestation day (GD) 0.5 to GD 16.5. In group two, dams were daily administered with the vehicle by oral gavage from GD 0.5 to GD 16.5. In group three, dams were injected intramuscular (IM) with NDTG at 25 mg/kg dose on GD 0.5 and GD 16.5. In group four, dams were injected a single IM injection of NDTG at 45 mg/kg on GD 0.5. T_1_ mapping, and embryo brain harvesting of DTG levels and proteomic assessments were completed on GD 17.5. **(E)** DTG concentrations in plasma of dams. DTG levels were measured at GD 16.5 to evaluate DTG levels in the mother’s blood. **(F)** DTG concentrations in whole brain tissues of embryos at GD 17.5. **(G)** DTG concentrations in placenta at GD 17.5. **(E–G)** Each sample (plasma or tissue) represents distinct litter. Data are expressed as mean ± SEM, N = minimum 3 animals/time point. *t*-test (two-tailed) was used (*p < 0.05, ^#^ *p* < 0.1).

Poloxamer 407 (P407)-DTG nanoformulations (NDTG) were prepared using high-pressure homogenization (Figure 1A). Direct synthesis approach was utilized (Sillman et al., 2018). Encapsulation efficiency of NDTG formulation was 82.7% (Figure 1B). Particle size, polydispersity index (PDI), and zeta potential for the nanoformulation were determined using dynamic light scattering (DLS). The size, PDI, and zeta potential of NDTG particles were 370 nm, 0.21, and −0.9  mV, respectively (Figure 1B). Further, scanning electron microscopy (SEM) was performed to determine morphology of nanoparticles. NDTG particles showed uniform, cuboidal- and rod-shaped morphologies (Figure 1C).

To assess NDTG effects on neurodevelopment, mice embryos were evaluated at gestation day (GD) 17.5 (Figure 1D). Pregnant C3H/HeJ female mice (dams) detected by vaginal plugs were randomly distributed in four study groups (Figure 1D). In group one, dams were orally administered every day with native DTG at human therapeutic equivalent dosage (5 mg/kg) by oral gavage starting at GD 0.5 up to GD 16.5. In group two, dams were treated orally every day with the vehicle used to prepare native DTG solution for oral administration (control) by oral gavage. Volume and administration scheme for vehicle was similar to native DTG oral administration. In group 3, dams were injected with two NDTG (25 mg/kg) intramuscular (IM) injections, first at GD 0.5 and second at GD 9.5. In group 4, dams were injected with a single intramuscular injection of NDTG (45 mg/kg) at GD 0.5. Periconceptional usage of DTG by women had more adverse pregnancy outcomes compared to drug initiation during pregnancy (Zash et al., 2018a; Zash et al., 2018b; Zash et al., 2019). Thus, DTG administration was started at the day of vaginal plug detection (GD 0.5) for all treatment groups (Figure 1D). This approach maintained parallel drug exposures among all pregnant dams. Blood collection from dams was completed at GD 16.5 to measure the drug concentrations in plasma. Maternal body weight gain during gestation was recorded and no significant differences were observed among vehicle (control), native DTG, and both NDTG (25 mg/kg or 45 mg/kg) groups (Supplementary Figure S1). At GD 17.5, T_1_ mapping was performed on live dams and embryo brain tissues were collected for global proteomics profiling and quantitation of DTG levels (Figure 1D).

Firstly, PK and BD profiles were determined for native-DTG and NDTG-treated groups. These tests were performed to test our hypothesis that injectable NDTG achieves therapeutic DTG concentrations in maternal blood and, at the same time, limits drug distribution to the embryo brain. DTG levels were measured in the plasma of dams at GD 16.5 and in the whole brain tissue of embryos and placental tissues at GD 17.5 (Figures 1E–G). Single (45 mg/kg) or two (25 mg/kg) IM injections of NDTG achieved equivalent plasma DTG levels to daily oral DTG administration (5 mg/kg) in pregnant dams (Figure 1E). In all the treatment groups, plasma DTG levels were between 4,000–6,500 ng/mL, which are comparable to therapeutic DTG concentrations from daily oral dosing in humans (Pain et al., 2015; Lewis et al., 2016; Mulligan et al., 2018; van der Galien et al., 2019). However, significantly lower levels of DTG biodistribution in embryo brain was observed following NDTG injections in comparison to daily oral administration (Figure 1F). For daily oral DTG administration, average DTG concentrations of 196 ng/g were recorded in the embryo brains compared to 34 ng/g and 45 ng/g for groups administered with single or two IM injections of NDTG, respectively. In addition, an average tissue concentration of 1,545 ng/g was recorded in placental tissue of the native DTG-treated group compared to 652 ng/g and 995 ng/g of DTG concentrations for single or two IM injections of NDTG, respectively (Figure 1G). Altogether, PK and BD data confirmed that LA DTG nanoformulations sustain therapeutic DTG levels in maternal plasma while limiting drug exposure to the embryo brain during pregnancy. The safety benefits of lower biodistribution of drug in embryo brain during gestation were validated in the succeeding *in utero* MRI and proteomics tests.

### NDTG prevents DTG-induced oxidative stress

**FIGURE 2:**
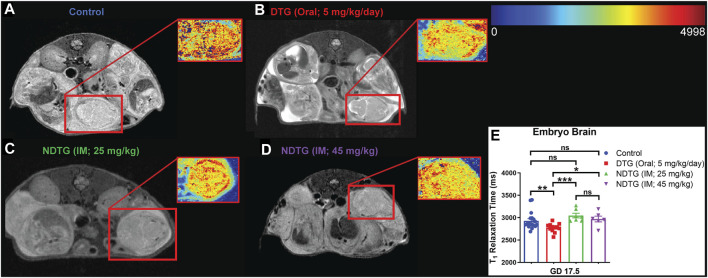
T_1_ mapping to examine T_1_-relaxivity of oxidative stress in embryo brain. **(A–E)** T_1_ mapping of embryo brain. Live dams were scanned at GD 17.5 to acquire T_1_ maps to examine T_1_-relaxivity of oxidative stress in embryo brain. T_1_ mapping was performed using RARE with varying TR = 120–7,500 ms, and TE = 32 ms and in-house IDL program was used for T_1_ fitting. Two embryos per dam were randomly selected for MRI scans. **(A–D)** Heat map and respective T_2_-weighted image of representative embryo brain from each group are encompassed by red boxes. **(E)** Comparison of T_1_ relaxation times among four study groups. N = minimum 6 animals/group. *t*-test (two-tailed) was used (*p < 0.05, **p < 0.01, ***p < 0.001).

Previously we demonstrated that native DTG treatment induces oxidative stress in the brains of adult mice (Montenegro-Burke et al., 2019). It has been shown that oxidative stress leads to a reduction in MRI T_1_ relaxation time (Tain et al., 2018; Tain et al., 2019). Therefore, to determine whether NDTG could ameliorate *in utero* DTG-induced oxidative stress in the embryo brain, live dams were scanned with a 7T MRI at GD 17.5 to acquire T_1_ maps to examine T_1_-relaxivity of oxidative stress in the embryo brain (Figures 2A–E). T_1_ mapping was performed using RARE sequence data with varying TR = 120–7,500 ms, and TE = 32 ms. In-house IDL program was used for T_1_ fitting at each pixel and generating T_1_ maps. The heatmaps of T_1_ relaxation times on the representative embryo brain from each study group are shown in Figures 2A–E. Representative embryo brains selected to quantitate T_1_ values from each study group are identified by a red square on a respective high-resolution T_2_-weighted MRI image. In the representative heatmap, decreased color intensity was observed in the embryo brains of the native DTG group compared to that in the embryo brains of vehicle-treated controls (Figures 2A,B). Remarkably, comparative color intensity was noted between control and either of the NDTG-treated groups (25 mg/kg or 45 mg/kg; Figures 2A, C, D). Moreover, higher color intensity was noted in brains of embryos of both NDTG groups than that in the embryo brain of the native-DTG group (Figures 2B–D). Further, average T_1_-relaxation times were calculated on T_1_ maps by ROI analysis using the MIPAV (Figure 2E). Significantly reduced T_1_ relaxation time was noted in native DTG-treated embryo brains compared to the control group, indicating DTG-induced oxidative stress. T_1_ relaxation times were significantly higher in embryo brains of both NDTG-treatment groups compared to those in embryo brains of the native-DTG treatment group. However, these observed T_1_ values were comparable between control and either of the NDTG-treatment groups. Similar T_1_ values between control and NDTG groups signified prevention of DTG-induced oxidative stress in the embryo brain. In addition, T_1_ values were comparable between both NDTG-treatment groups. Overall, T_1_ mapping suggested that lower drug exposure to the embryo brain using injectable LA DTG nanoformulations can prevent native drug induced oxidative stress.

### NDTG attenuates DTG-induced developmental neuronal impairments

**FIGURE 3:**
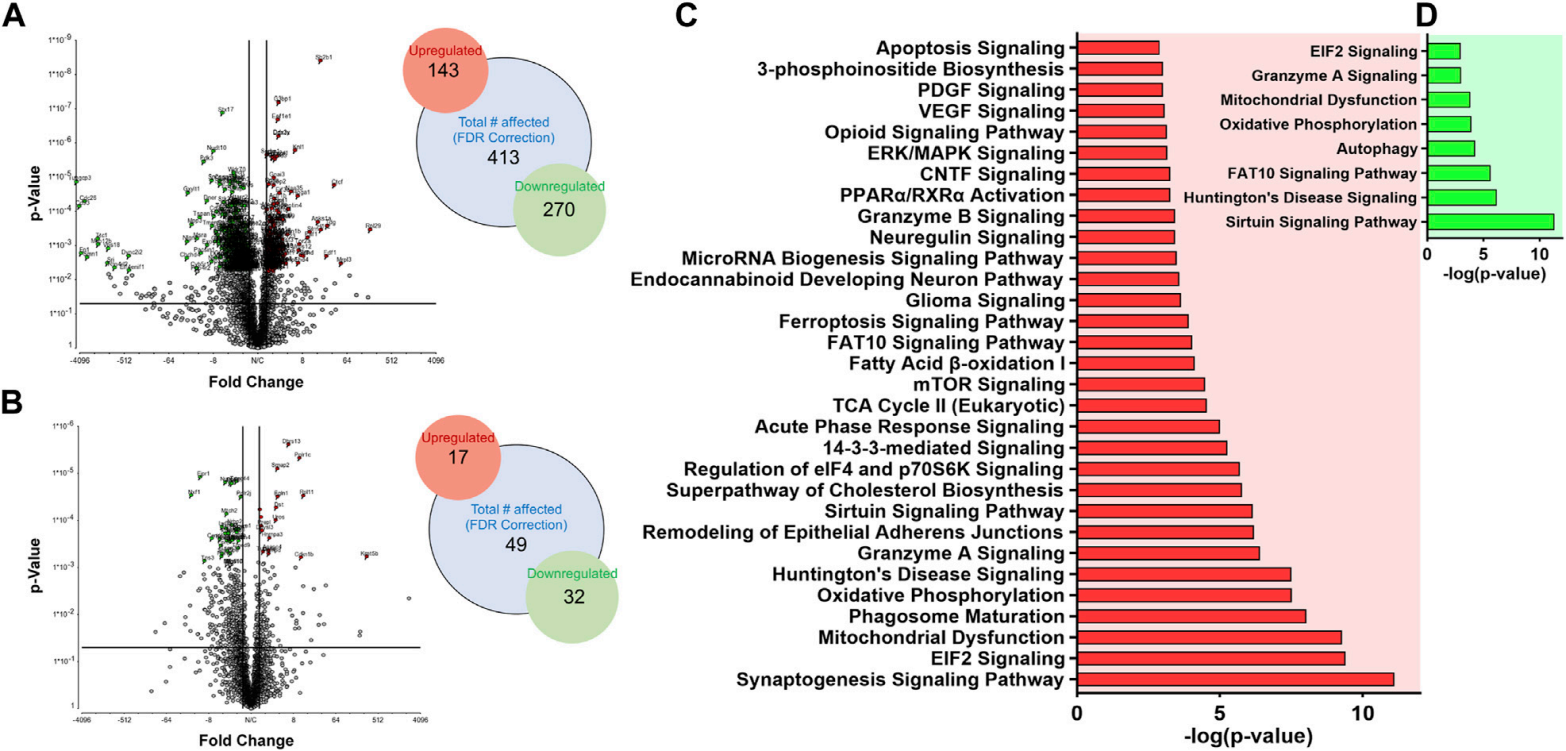
Proteomic profiling of embryos brains following DTG or NDTG treatment. Non-targeted proteomic profiling of embryo whole brain tissues. Comparison between control and native DTG groups or control and NDTG groups were performed. Control: N = 7 animals; native DTG (oral): N = 9 animals; NDTG: N = 5 animals. **(A,B)** Volcano plot of significantly altered proteins after FDR correction. Red color: Upregulated proteins; Green color: Downregulated proteins; *p* ≤ 0.05; Absolute Fold Change ≥2 or ≤ −2. Schematic presentation of total number of affected proteins including upregulated and downregulated proteins. **(C,D)** Major affected canonical pathways were determined by using Ingenuity pathway analysis (IPA). Comparison was performed between **(C)** control and native DTG groups or **(D)** control and NDTG groups.

After T_1_ mapping of live dams, embryo brain tissues were harvested at GD 17.5, and randomly selected whole brain tissues from embryos from distinct dams of each group were processed for non-targeted proteomic tests to evaluate the effects of DTG or NDTG exposure during gestation on developmental neurobiological pathways (Figure 1D). Comparisons of protein changes were performed between controls and native DTG (oral) or controls and NDTG. T_1_ relaxation times on embryos brains calculated from T_1_ maps and DTG concentrations in embryos brains were comparable between both NDTG groups (Figures 1F, 2F). Thus, for proteomic profiling, to justify sample size and cost-effectiveness, whole brain samples from both NDTG groups were combined and were identified as a single NDTG group. After false discovery rate (FDR) correction, comparison of proteins between the native DTG-treated and vehicle-treated group (control) showed a total of 413 proteins significantly differentially expressed, out of which 270 proteins were downregulated and 143 were upregulated (Figure 3A). Whereas comparison of proteins between the NDTG-treated and control groups showed a total of only 49 proteins significantly differentially expressed, out of which 32 proteins were downregulated and 17 were upregulated (Figure 3B). PCA analysis of proteins showed 53.2% and 53.1% distribution for DTG vs. control and NDTG vs. control comparisons, respectively, confirming differences between treatment and control groups (Supplementary Figures S2A, B). Further analysis using ingenuity pathway analysis (IPA) revealed that differentially expressed proteins were majorly associated with pathological canonical pathways including mitochondrial dysfunction, reduced energy production, increased cell damage or death, and impaired neuronal or synaptic development in the embryo brains of the native DTG group compared to controls (Figure 3C). The top 30 affected canonical pathways in the native DTG group in comparison to controls are shown in Figure 3C. Special mentions include mitochondrial dysfunction pathway, oxidative phosphorylation, EIF2 signaling, granzyme A signaling, phagosome maturation, sirtuin signaling, synaptogenesis signaling, and endocannabinoid developing neuron pathway. Whereas changes in these pathways were not detected or were attenuated in the NDTG treated group in comparison with controls (Figure 3D).

**FIGURE 4:**
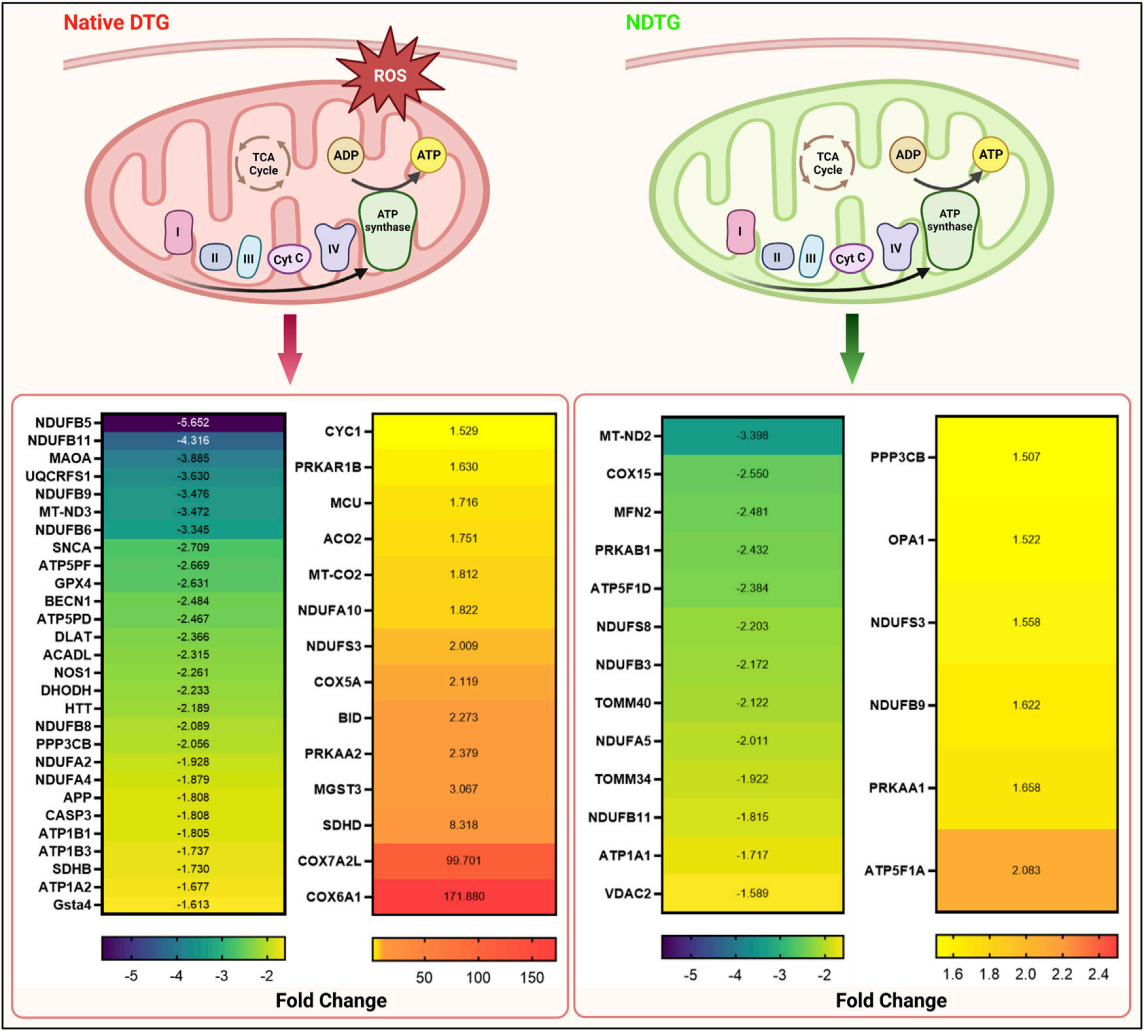
NDTG attenuates DTG-induced proteomic changes linked to mitochondrial dysfunction**.** Differentially expressed genes associated with mitochondrial dysfunction were determined by using IPA. Comparisons were performed between control and native DTG groups (red colored mitochondria, left side of the figure) or control and NDTG groups (green colored mitochondria, right side of the figure). Significantly differentially expressed genes were presented in heat maps. Control: N = 7 animals; native DTG (oral): N = 9 animals; NDTG: N = 5 animals.

**FIGURE 5:**
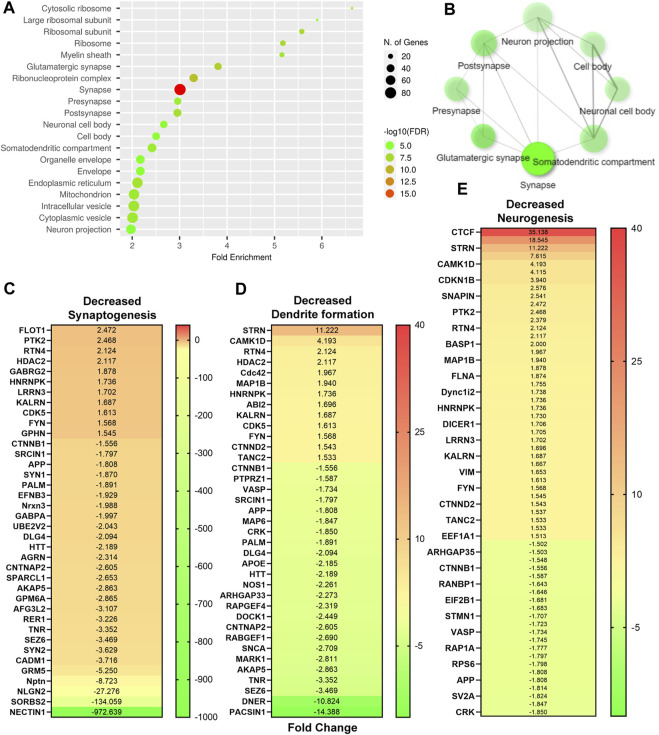
Native DTG-linked developmental neuronal deficits**. (A,B)** Enrichment analysis of significantly altered proteins after FDR correction was completed using ShinyGo. Comparisons were performed between control and native DTG groups. DTG-exposure affected neuronal components were detected using GO-cellular components annotation. **(C–E)** Significantly differentially expressed genes associated with (**C**) synaptogenesis, (**D**) dendrite formation, and **(E)** neurogenesis were determined using IPA and presented in heat maps. Control: N = 7 animals; native DTG (oral): N = 9 animals.

After identification of native-DTG induced oxidative stress in the embryo brain and of therapeutic benefits of injectable long-acting nanoformulations to prevent drug-induced oxidative stress, further analysis was performed using IPA for biological validation of MRI data. Comparative assessment with controls found that differentially expressed proteins in the native-DTG-treated group were linked to mitochondrial dysfunction and oxidative stress (Figure 4 and Supplementary Figure S3A). In the native-DTG-treated group a total of 42 proteins associated with mitochondrial function were significantly differentially expressed compared to controls. Comparatively, only 19 proteins associated with mitochondrial function were differentially expressed in the NDTG-treated group. In the native-DTG-treated group, proteins linked to the electron transport chain were NDUFA2, NDUFA4, NDUFA10, NDUFB5, NDUFB6, NDUFB8, NDUFB9, NDUFB11, NDUFS3, MT-ND3 (Complex I), SDHB, SDHD (Complex II), UQCRFS1, CYC1 (Complex III), and MT-CO2, COX5A, COX6A1 (Complex IV). In addition, depletion of energy production was indicated by downregulated ATP5PF, ATP5PD (Complex V). Other affected proteins were those associated with antioxidant properties (GPX4) and required for mitochondrial homeostasis (DLAT, ACADL, NOS1, MAOA, DHODH). Interestingly, these differentially expressed proteins associated with mitochondrial dysfunction were ameliorated in NDTG-treated group (Figure 4 and Supplementary Figure S3B). The list of proteins and fold change differences are shown in heat maps (Figure 4). Overall, proteomic data validated the MRI T_1_ mapping data, signifying prevention of DTG-induced oxidative stress when delivered as LA nanoformulations. Further analysis using ShinyGo and IPA revealed that differentially expressed proteins were majorly associated with developmental cellular impairments including decreased neurogenesis and synaptogenesis, and aberrant neuronal growth in the embryo brains of the native DTG group compared to controls (Figures 5A,B and Supplementary Figure S4). Whereas these detrimental pathways were not detected in the NDTG treated group compared to controls (Supplementary Figure S5). Differentially expressed proteins served to confirm impairment in neurogenesis, synaptogenesis and neurite formation in the native-DTG-treated group compared to control group. These proteins and their expression levels are presented with heatmaps (Figures 5C–E). Altogether, non-targeted proteomic assessments confirmed that delivery of DTG as an injectable LA nanoformulation can prevent native DTG-linked impairment of neuronal development.

### *In utero* NDTG or native-DTG exposure did not cause neural tube defects (NTDs)

To determine the effect of native DTG or NDTG administration during pregnancy on embryo structural development, embryos were harvested and evaluated for NTDs at GD 17.5. Embryo phenotypes in native DTG, NDTG (25 mg/kg or 45 mg/kg) or vehicle treatment groups are summarized in Table 1. No loss of pregnancy was noted in any of the treatment groups. The litter size, viability and resorption rates were similar between groups. The mean litter size in control, native DTG (5 mg/kg/day; oral), NDTG (25 mg/kg; IM) and NDTG (45 mg/kg; IM) group was 6, 8, 8, and 6.6 respectively. The total resorptions (%) in vehicle-treated dams (control) was 18.7% (n = 8 litters, 9 out of 48 total implants); in native DTG-treated dams was 12.5% (n = 6 litters, 6 out of 48 total implants); in NDTG (25 mg/kg)-treated dams was 9.3% (n = 4 litters, 3 out of 32 total implants); and in NDTG (45 mg/kg)-treated dams was 25% (n = 3 litters, 5 out of 20 total implants). No NTDs were detected (0%) in any of the study groups (Table 1). Taken together, *in utero* DTG exposures through oral DTG or IM NDTG did not lead to fetal NTDs.

**TABLE 1:** Embryo phenotypes.

	Vehicle	DTG	NDTG	NDTG
	Oral	(5 mg/kg/day) oral	(25 mg/kg) 2 × IM	(45 mg/kg) 1 × IM
Total litters	8	6	4	3
Viable pregnancy (%)	100%	100%	100%	100%
Litter Means				
** **Implants/litter	6	8	8	6.6
** **Viable embryos/litter	4.8	7	7.2	5
** **Resorptions/litter	1.1	1	0.7	1.6
** **Abnormal/litter	0	0	0	0
Embryo Phenotype (%)				
** **Total implants	48	48	32	20
** **Total resorptions	9 (18.7%)	6 (12.5%)	3 (9.3%)	5 (25%)
** **Total viable	39 (81.25%)	42 (87.5%)	29 (90.6%)	15 (75%)
Viable Embryos (%)				
** **Normal	39 (100%)	42 (100%)	29 (100%)	15 (100%)

## Discussion

Transitioning of a majority of RLCs to DTG-based generic regimens as a recommended first-line therapy is already in place. This is essentially due to advantages over other ARV-regimens, which include high potency, barrier to resistance, and cost-effectiveness. Currently, RLCs hold more than two-thirds of the world’s total HIV-1 infected population (The Joint United Nations Programme on HIV/AIDS UNAIDS, 2021). Thus, usage of a DTG-based regimen by most pregnant women LWHIV or those of child-bearing age worldwide will be inevitable. With the recurring acknowledgment from clinical and basic science research about potential associations of DTG with neurodevelopmental toxicity (Zash et al., 2018a; Cabrera et al., 2019; Zash et al., 2019; Crowell et al., 2020; Mohan et al., 2020; Bade et al., 2021; Kirkwood-Johnson et al., 2021; Tukeman et al., 2023), the development of novel therapeutic interventions to maximize therapeutic gains of highly efficacious DTG while limiting adverse effects during pregnancy is timely. Recently, clinical usage of LA-CAB for treatment and prevention therapies (U S Food and Drug Administration FDA, 2021a; U S Food and Drug Administration FDA, 2021b) has emerged. Thus, with the suitable physiochemical properties of DTG for the transformation into LA formulation schemes (Sillman et al., 2018; Deodhar et al., 2022), development of LA-DTG with improved safety and therapeutic profiles during pregnancy provides novel delivery means with translational potential. With this goal in mind, we developed LA poloxamer coated NDTG and evaluated its safety and benefits against daily oral native DTG administration in pregnant mice. Our preliminary works, for the first time, demonstrated that LA-DTG nanoformulations attenuate native DTG-associated impairment of neuronal development in the embryo brain. We hypothesize that the neuroprotective effect is the outcome of lower drug biodistribution in the embryo brain during gestation following NDTG treatment in comparison to daily oral DTG administration. Detected lower drug biodistribution in embryos brains is predicted to be due to the pharmacological properties of LA formulations. These include a longer half-life, maintenance of long-term lower C_max_, and enhanced bioavailability. This is possible due to sustained release of DTG from formulations and tissue depots of formulations (Montenegro-Burke et al., 2018; Sillman et al., 2018). Due to these key LA properties, there is a reduction in the total amount of native drug exposure over time compared to daily administration. For example, daily oral administration of clinically approved CAB (VOCABRIA) at 30 mg dose will end up with 1,680 mg of drug intake in 8 weeks. Whereas dosage of a single IM injection of bi-monthly LA-CAB (CABENUVA or APRETUDE) is 600 mg. These dosage differences result in an approximately 3-fold reduction in total amount of native drug exposure through LA formulation compared to daily oral administration with equivalent duration of action (U S Food and Drug Administration FDA, 2021a; U S Food and Drug Administration FDA, 2021b).

In addition to lower drug exposure, poloxamer surfactant in the nanoformulations provides supplemental therapeutic benefits. Poloxamers are neuroprotective, anti-inflammatory, and antioxidants, and are well known to promote resealing of disrupted plasma membranes (Curry et al., 2004a; Curry et al., 2004b; Serbest et al., 2006; Cadichon et al., 2007; Dalal and Lee, 2008; Moloughney et al., 2012; Luo et al., 2013; Luo et al., 2015). In the current work, P407 was used as a surfactant. Choice of P407 was based on our own work that previously showed the benefits of LA P407 encapsulated DTG nanoformulations in ameliorating the DTG-associated oxidative stress in the central nervous system (CNS) of adult mice (Montenegro-Burke et al., 2018). This was confirmed using the global metabolomics assessments in various brain regions of adult mice. Of translational significance, toxicology studies have shown that poloxamers are safe at very high concentrations and were approved by the US FDA for pharmaceutical products (Singh-Joy and McLain, 2008). Among widely used surfactants, other research groups have noted that P188 has neuroprotective benefits (Curry et al., 2004a; Curry et al., 2004b; Serbest et al., 2006; Cadichon et al., 2007; Dalal and Lee, 2008; Moloughney et al., 2012; Luo et al., 2013; Luo et al., 2015). Moreover, polymer surfactants, such as poloxamer 338 and polyethylene glycol 3,350, are utilized in clinical LA-ARV injectable formulations (Trezza et al., 2015; Williams et al., 2015). Thus, screening of additional poloxamer-based LA nanoformulations of DTG will be needed in the future to determine comparative therapeutic benefits for neurodevelopmental outcomes.

The notion of nanoformulations usage to increase efficacy and safety of drugs used to treat other diseases during pregnancy has been raised, which underscores our LA platform for improved safety of antiretrovirals in HIV-1 infected pregnant women (Zhang et al., 2019; Pritchard et al., 2021). Importantly, our data confirmed that NDTG also maintained comparable therapeutic DTG levels in dams’ blood during gestation. Sustaining such therapeutic levels in the mother is essential to control viral replication (Trezza et al., 2015; Williams et al., 2015; Sillman et al., 2018). Moreover, it can help to prevent known adverse effects of DTG on females such as weight gain and neuropsychiatric adverse events (Hoffmann et al., 2017; Venter et al., 2019; Caniglia et al., 2020; Lake et al., 2020; Eifa and Ketema, 2022), providing additional benefits to limit indirect adverse effects through maternal morbidity on fetal neurodevelopment (Yombi, 2018). Even though combinational therapy is required for treatment regimens, development of single ARV nanoformulations justifies clinical relevance. Currently, for maintenance therapy of HIV-1 infected people, separate injections of each ARV LA nanoformulations are given at the same time. Specifically, two distinct LA-formulation injections of CAB and rilpivirine (RPV) are clinically used for maintenance therapy (U S Food and Drug Administration FDA, 2021a; U S Food and Drug Administration FDA, 2021b). Despite the fact that DTG is currently not recommended for prevention therapy, usage of monotherapy of LA-CAB (same antiretroviral class; INSTIs) injection for prevention (U S Food and Drug Administration FDA, 2021b) and successful reports from basic science research showing potential application of LA-DTG for prevention in research animals (Sillman et al., 2018), development and testing of the LA-DTG formulation is timely and could benefit both treatment and prevention settings. We acknowledge that rodents and humans present different PK profile for therapeutic drugs due to, but not limited to, variation in absorption, distribution, and metabolism mechanisms. However, association of detected drug levels in research animals with clinically relevant therapeutic levels is essential. Herein, we noted that P407-DTG formulations maintain clinically relevant therapeutic drug levels (Pain et al., 2015; Lewis et al., 2016; Mulligan et al., 2018; van der Galien et al., 2019) in pregnant mice plasma during gestation. Previously, we showed that P407-DTG formulation at a 45 mg/kg dosage maintains therapeutic drug levels up to a month following a single IM injection in non-pregnant adult male mice (Sillman et al., 2018). Thus, single injection of NDTG at a 45 mg/kg dosage (mouse weight) at the day of plug detection will lead to a total of 1.12 mg of drug exposure compared to 2.5 mg of total drug exposure following daily oral native DTG administration at a clinically relevant therapeutic dose of 5 mg/kg during approximately 20 days of gestation in a mouse. This represents an approximately 2-fold reduction in drug exposure with LA DTG. Indeed, current PK data are from rodents, however, due to current clinical usage of LA formulations of ARVs, established neuroprotective properties and safety of poloxamers, and potential reduction in drug exposure to embryo while maintaining therapeutic levels in mother due to pharmacological properties of LA formulations, our data provide a novel tool to maximize therapeutic benefits of DTG during pregnancy.

We did not observe any type of NTDs in native DTG or NDTG-treatment groups. Although no NTDs were observed in NDTG-treated groups (25 or 45 mg/kg), sample size was limited to 3 to 4 animals per group. Thus, further assessment with a larger sample size per group is required for conclusive remarks linked to LA formulations. However, the most updated data from the Tsepamo study from Botswana showed a declined rate of native DTG-linked birth defects and comparative numbers between DTG and other ARVs at the time of conception (the 24th International AIDS Conference, 2022). This is similar to our observations of low NTDs following native DTG exposure at conception in the current and previous work at therapeutic and supratherapeutic dosages, respectively (Bade et al., 2021).

Fetal neurodevelopmental mechanisms are highly susceptible to certain chemical exposures during gestation. Thus, DTG readily reaching to the embryo CNS throughout the gestational period can potentially cause physiological, metabolic, or functional harm to the brain and may increase risk for postnatal neurodevelopmental deficits. Our current and previous works (Bade et al., 2021) confirmed the exposure of embryos brains to DTG during gestation in mice. Clinical studies also found high placental transfer of DTG from mother to fetus. Reported median ratios of drug levels in cord blood to maternal blood ranged between 1.21 and 1.29 (Mulligan et al., 2018; Waitt et al., 2019; Bollen et al., 2021). In addition, DTG accumulation in fetuses was confirmed with prolonged elimination from infants following gestational transfer (Mulligan et al., 2018; Waitt et al., 2019). Transplacental transfer of DTG at efficient levels to the fetus can potentially protect it from HIV-1 infection. However, at the same time, the fetus is exposed to the drug during a highly critical period of neurodevelopment, which may lead to drug-induced neurodevelopmental impairments. In the current work, non-targeted proteomic profiling and T_1_ mapping identified native-DTG induced dysregulations of proteins linked to mitochondrial dysfunction, oxidative stress, and impaired neuronal development. Different biological mechanisms have been identified to be affected by DTG which could lead to observed neurodevelopmental toxicity. Previously, our works demonstrated that DTG-induced inhibition of MMPs activities in mice embryo brain during gestation can affect postnatal neurodevelopment (Bade et al., 2021). This was noted with neuronal impairment and neuroinflammation during early postnatal evaluation of brain health of mice pups following *in utero* DTG exposure. In addition, Daniela Zizioli et al. and others observed decreased expression of crucial genes, *neurog1* and *neurod1,* required for early neuronal differentiation following CAB exposure in zebrafish embryos, suggesting decreased or delayed neurogenesis (Zizioli et al., 2023). Due to chemical structural similarities among integrase strand transfer inhibitors (INSTIs), observations on CAB-linked decreased neurogenesis could be applied to our observations of DTG exposure on decreased neurogenesis and development. Currently, there are no other pre-clinical studies which have evaluated direct effects of DTG and other INSTIs on functional or molecular neurodevelopment following usage during pregnancy or studied linked patho-biological mechanisms underlying potential adverse events. Thus, future research efforts are required to identify unknown adverse events on the developing brain and understand underlying mechanisms.

In recent years, significant efforts from clinical and basic science research groups were engaged to study DTG-associated fetal structural defects and potential underlying mechanisms after an interim analysis of the Tsepamo study showed an increased risk of NTDs in infants following maternal periconceptional usage of DTG (Zash et al., 2018a; Cabrera et al., 2019; Zash et al., 2019; Mohan et al., 2020; Kirkwood-Johnson et al., 2021; Tukeman et al., 2023). Yet there is a knowledge gap of adverse events reflecting DTG-associated postnatal neurodevelopmental impairments. This is essential for babies born without structural malformations. It remains a major research gap, especially when the risk of NTDs is very low (0.2%–0.3%) or comparable to other ARVs and most infants are, or will be, potentially born without structural malformations after DTG exposure. Indeed, it is now well accepted that DTG causes neuropsychiatric adverse events in adults ranging from insomnia to severe depression (Yombi, 2018; Amusan et al., 2020). DTG-associated neuronal toxicity is also confirmed by *in vitro* and animal studies (Montenegro-Burke et al., 2019; Smith et al., 2022). Importantly, our findings showed the potential adverse effect of DTG on postnatal neurodevelopment due to direct adverse impacts on neuronal development during gestation. Recently, the SMARTT study reported an increased risk of postnatal neurological abnormalities during development such as febrile seizures, microcephaly, epilepsy and other neurological disorders following gestational exposure (Crowell et al., 2020). Therefore, with a large number of fetuses being exposed to DTG worldwide, continuous research efforts are critical to uncover any adverse effects of DTG exposures on pre- or post-natal neurodevelopment and to determine underlying mechanisms so that intervention strategies can be developed. Importantly, scientific exchange between basic science mechanistic findings and the clinical assessment of DTG-exposed children will be required in the future. Such approach will cross-validate underlying mechanisms, assess clinical relevance of scientific findings, and provide rigorous assessments of neurodevelopmental outcomes. Altogether, it is timely to identify any potential DTG-induced secondary effects during pregnancy to provide effective care for women LWHIV and their children.

Although the current study provides evidence of improved safety of DTG when administered as LA-DTG formulations compared to daily oral native DTG during pregnancy, it was limited by sample size, different administration time points from pre-conception and to during gestation, and various dosages. Comprehensive PK and BD assessments will be needed in the future to determine precise drug distribution to fetuses and identify links to the safety. Moreover, LA formulations with different DTG apparent half-lives need to be investigated. Notably, prodrug-based LA formulations of INSTIs have shown potential to maintain therapeutic levels of drugs up to 6 months or longer duration. Thus, assessments of these LA formulations of DTG-prodrugs with neuroprotective surfactants for improved safety remains our own research interest. Lack of assessment of steady state drug levels of LA formulations during pregnancy due to the shorter gestational period of mice remains a question for future investigation. Another limitation of the current study is the absence of an IM poloxamer (P407) only group as a control. In addition, mothers on DTG-based regimens are HIV-1 positive, and such maternal infection, even at an undetectable level, could be a potential confounding factor. However, normal mice do not get infected with HIV-1; and humanized mice developed for HIV-1 infection cannot breed, thus, to establish a cause-and-effect relationship with a single agent, PK and adverse effects were evaluated in normal mice. Nonetheless, sustained therapeutic levels in dams which are equivalent to clinical values addresses concerns related to drug levels required for viral control in the mother. Also, we previously showed the prevention and treatment benefits from sustained therapeutic levels of DTG (Sillman et al., 2018). Future studies are necessary in order to affirm pharmacological and mechanistic links between reduced drug exposure and neurodevelopmental outcomes. Overall, our preliminary works showed that LA DTG nanoformulations potentially provide (World Health Organization WHO, 2016) sustained therapeutic levels of DTG in the mother’s blood (U.S. Department of Health and Human Services, 2023), improved neurodevelopmental outcomes by reducing DTG exposure to embryo brain due to reduced total drug intake over time compared to daily oral pills and providing supplemental benefits of poloxamer surfactants.

## Data Availability

The datasets presented in this study can be found in online repositories. The names of the repository/repositories and accession number(s) can be found below: https://figshare.com/articles/dataset/Proteomics_DTG_vs_Control_and_NDTG_vs_Control_Significantly_changed_Proteins_after_FDR_correction_csv/24150246.
