# Supplementary material for: Long-acting dolutegravir formulations prevent neurodevelopmental impairments in a mouse model
Source: Front Pharmacol. 2023 Dec 12;14:1294579. doi: 10.3389/fphar.2023.1294579 (PMC10750158; doi:10.3389/fphar.2023.1294579)
Supplement: Supplementary file 1 [file DataSheet1.DOCX]

**Supplementary Material**

**Long-acting Dolutegravir Formulations Prevent**

**Neurodevelopmental Impairments in a Mouse Model**

Emma G. Foster^1^, Brady Sillman^1^, Yutong Liu^2^, Micah Summerlin^1^, Vikas Kumar^3^,

Balasrinivasa R. Sajja^2^, Adam R. Cassidy^4^, Benson Edagwa^1^,

Howard E. Gendelman^1,5^, Aditya N. Bade^1^*

^1^Department of Pharmacology and Experimental Neuroscience, University of Nebraska Medical Center, Omaha, NE 68198, USA.

^2^Department of Radiology, University of Nebraska Medical Center, Omaha, NE 68198, USA.

^3^Department of Genetics, Cell Biology, and Anatomy, University of Nebraska Medical Center, Omaha, NE 68198, USA.

^4^Departments of Psychiatry and Psychology & Pediatric and Adolescent Medicine, Mayo Clinic, Rochester, MN 55905, USA.

^5^Department of Pharmaceutical Sciences, University of Nebraska Medical Center, Omaha, NE 68198, USA.

***Corresponding author:** Aditya N. Bade, Ph.D., Department of Pharmacology and Experimental Neuroscience, University of Nebraska Medical Center, Omaha, NE 68198-5800, USA; phone: 402-559-4050; fax: 402-559-7495; email: [aditya.bade@unmc.edu](mailto:aditya.bade@unmc.edu), ORCID: <https://orcid.org/0000-0003-2511-4461>

**Supplementary Data**





**Supplementary Figure 1. Maternal body weight gain during gestation.** Weight gain of dams was recorded during gestation and no significant differences were observed among control, DTG, and NDTG groups.


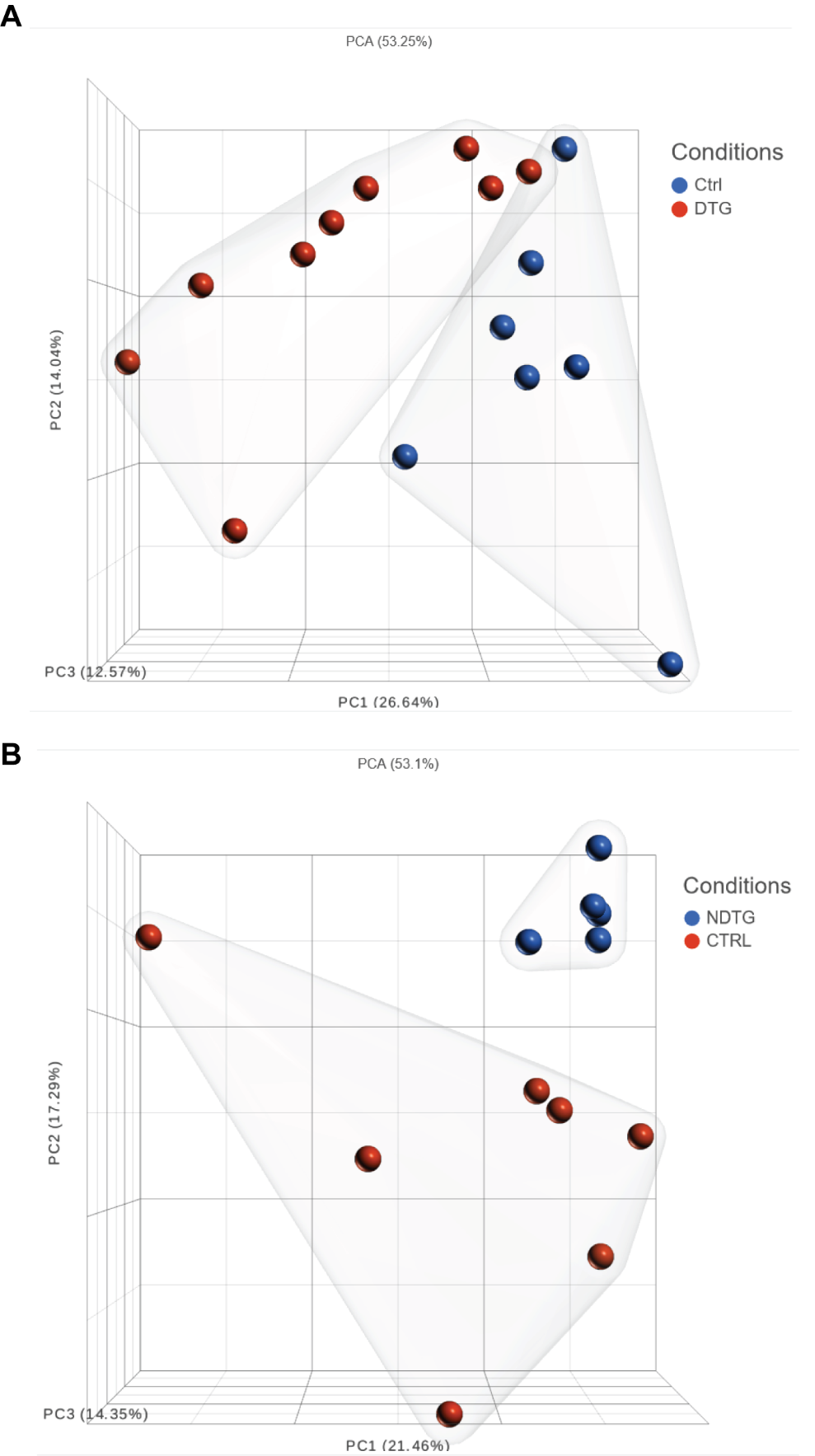


**Supplementary Figure 2. Principal component analysis (PCA) of proteins.** PCA plots were generated using Partek Genomics Suite 7.0. (**A-B**) PCA analysis of proteins shows (**A**) 53.2% distribution variance for control vs DTG and (**B**) 53.1% distribution variance for control vs NDTG. Control: N = 7 animals; native DTG (oral): N = 9 animals; NDTG: N = 5 animals.


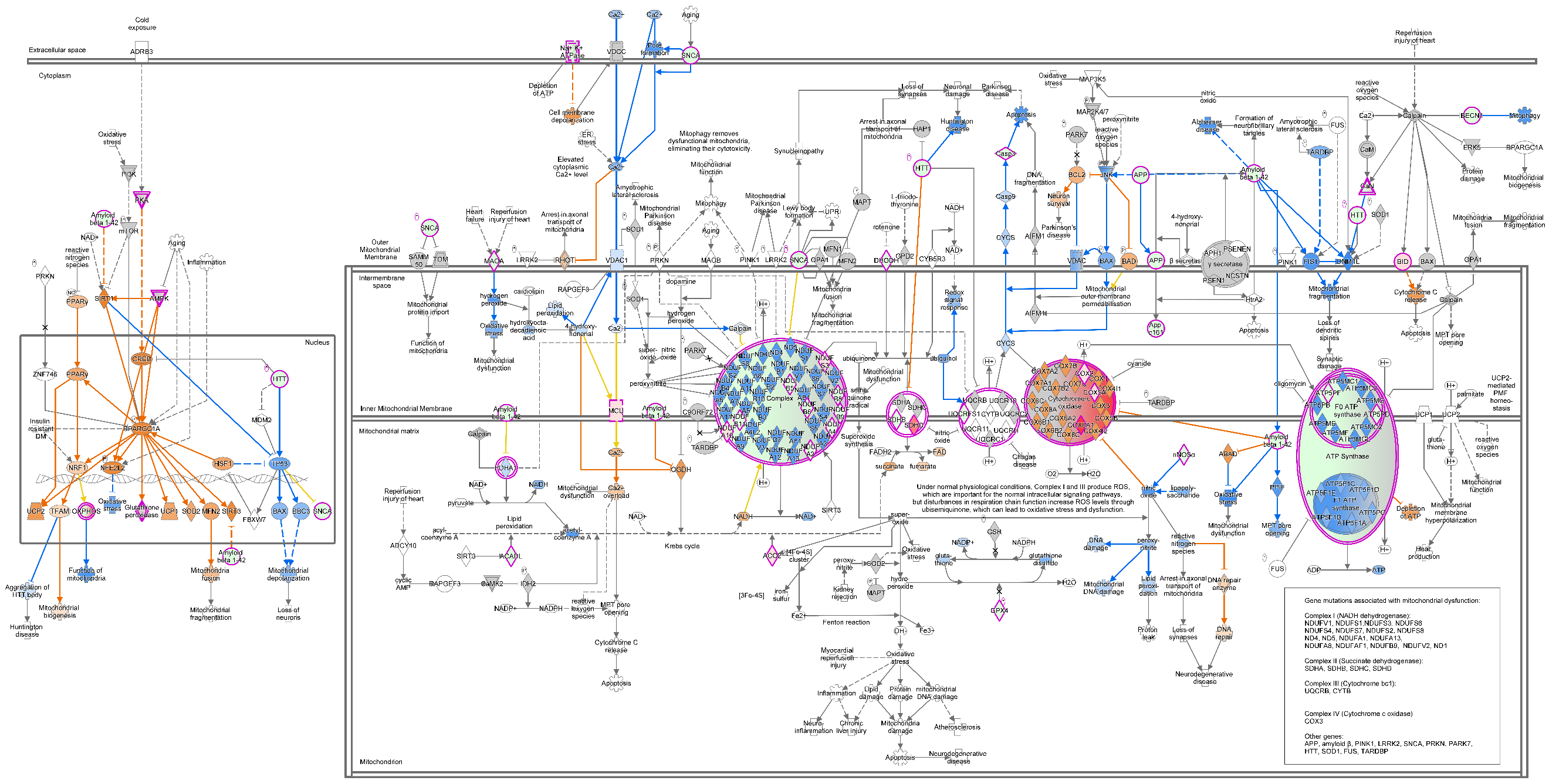


Cytoplasm

Mitochondrion

Extracellular space

**A**

**DTG**


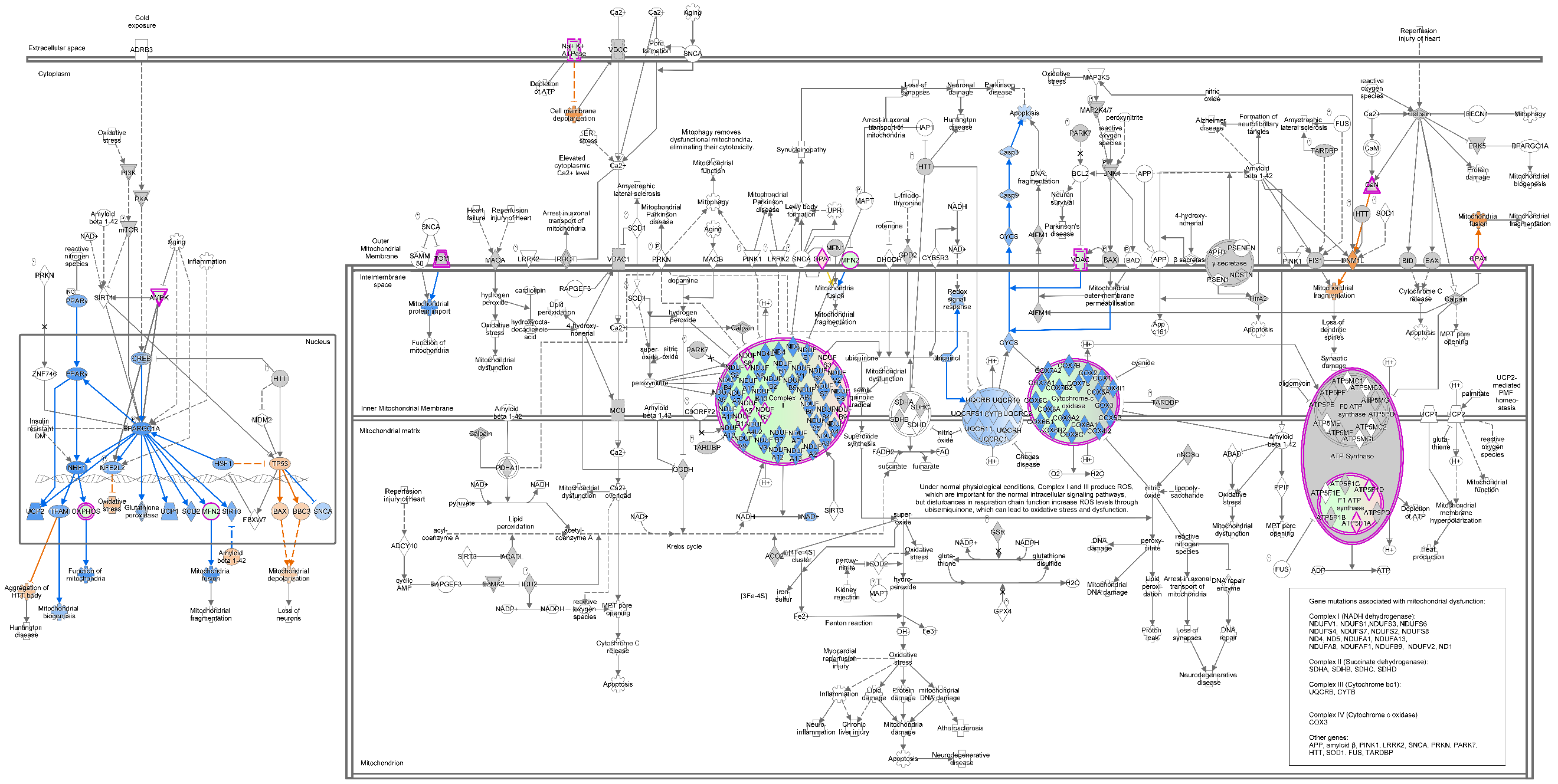


Mitochondrion

Cytoplasm

Extracellular space

**B**

**NDTG**

**Supplementary Figure 3. Differentially expressed proteins associated with mitochondrial dysfunction and oxidative stress.** (**A-B**) Differentially expressed genes associated with mitochondrial dysfunction were determined by using IPA. (**A**) Comparative assessment between control and DTG revealed differentially expressed proteins linked to mitochondrial dysfunction and oxidative stress. **(B)** Comparative assessment between control and NDTG revealed an amelioration of differentially expressed proteins associated with mitochondrial dysfunction. Pathway figure was generated using IPA. Red color: increased measurements; Green color: decreased measurements; Blue color: predicted inhibition; Orange color: predicted inhibition; Orange solid line: leads to activation; Blue solid line: leads to inhibition; Yellow solid line: Findings inconsistent with state of downstream molecule; Black solid line: effect not predicted; dashed line: indirect relationship; and solid line: direct relationship. Control: N = 7 animals; native DTG (oral): N = 9 animals; NDTG: N = 5 animals.


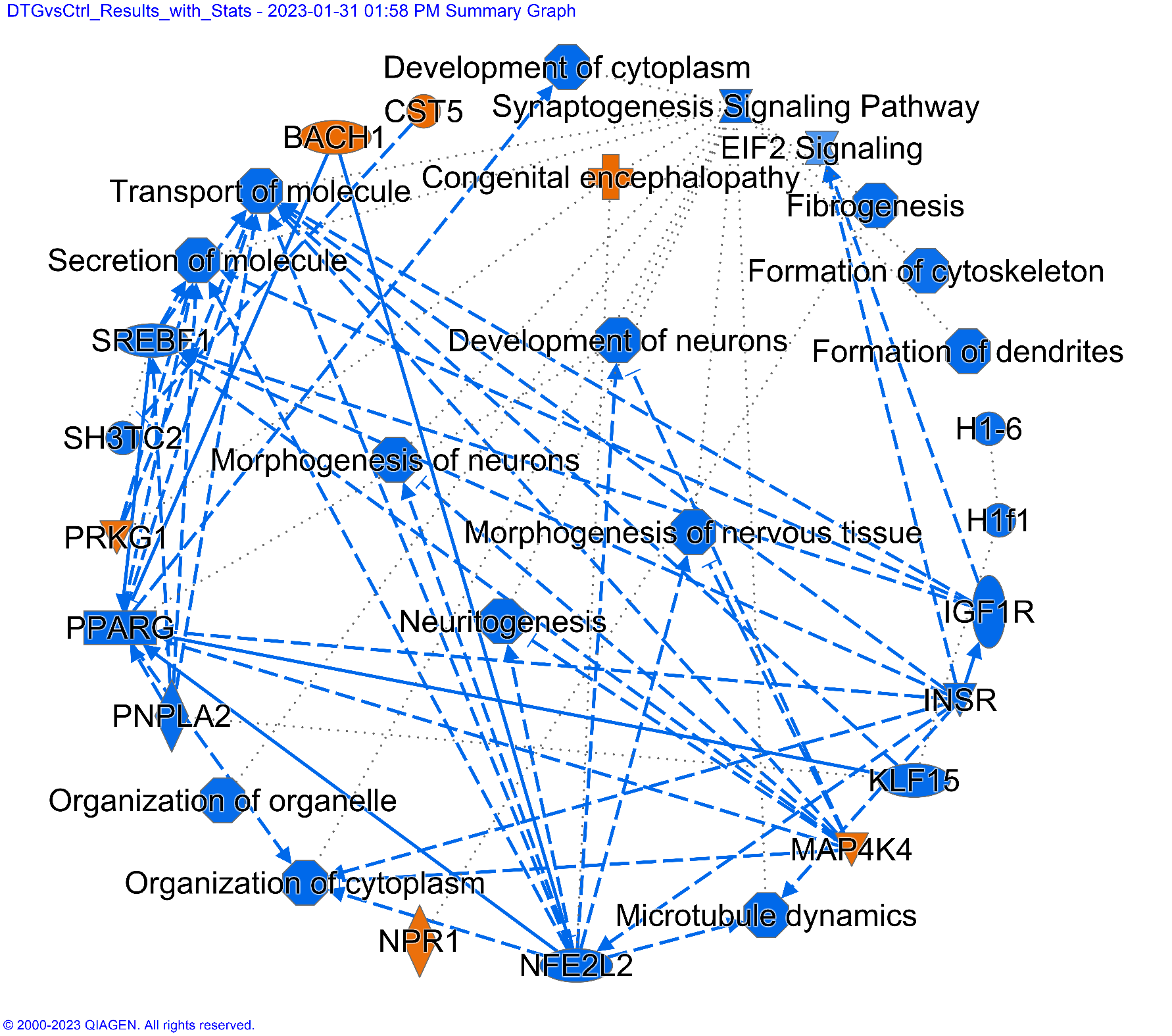


**Supplementary Figure 4. Native DTG-associated developmental neuronal impairments in embryo brain.** IPA analysis of differentially expressed proteins in native DTG-treated embryo brains compared to controls reveal decreased neurogenesis and aberrant neuronal growth (center of the figure). Figure was generated using IPA. Blue color: predicted inhibition; Orange color: predicted inhibition; Orange solid line: leads to activation; Blue solid line: leads to activation; Solid line: direct interaction; Intermittent line: indirect interaction; and dotted line: inferred relationship. Control: N = 7 animals; native DTG (oral): N = 9 animals


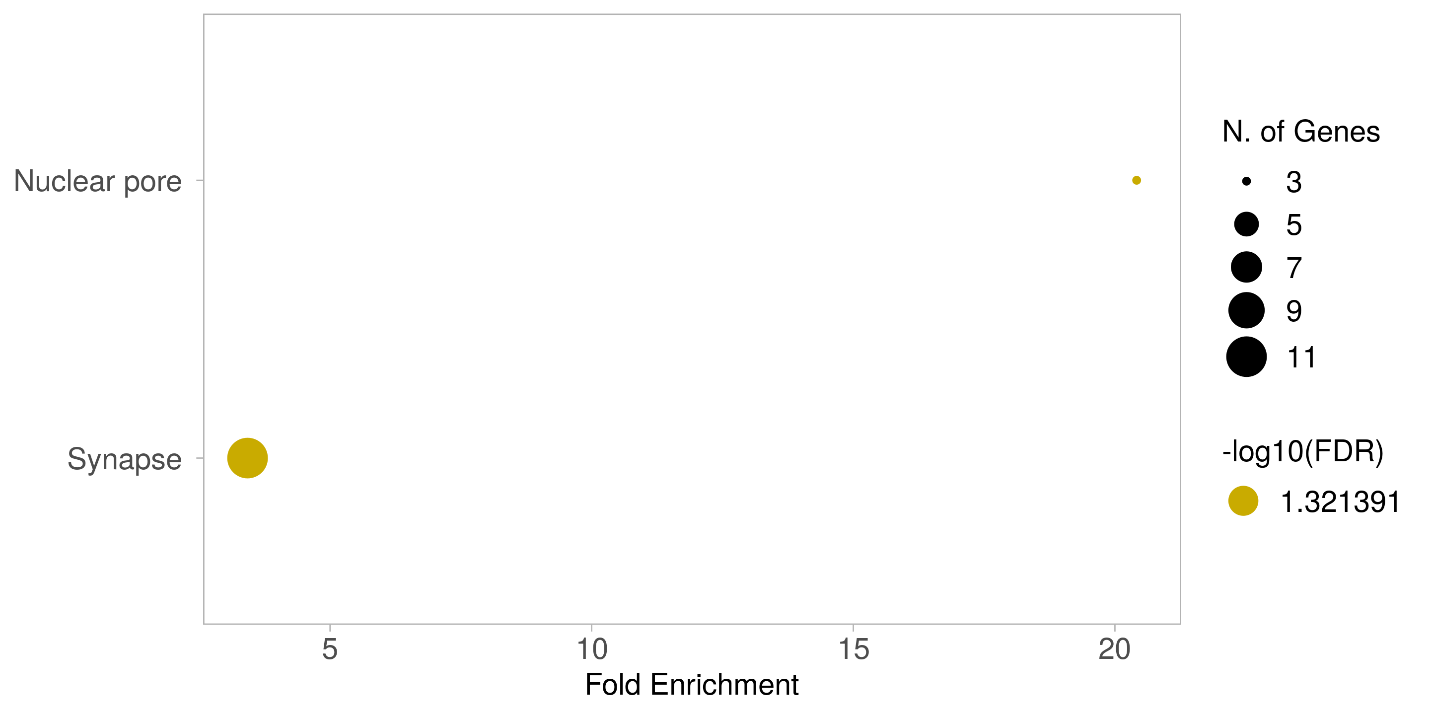


**Supplementary Figure 5. NDTG ameliorates DTG-induced protein changes linked to neuronal impairments.** Enrichment analysis of significantly altered proteins after FDR correction was completed using ShinyGo. Comparisons were performed between control and NDTG groups. DTG-exposure affected neuronal components were detected using GO-cellular components annotation. Control: N = 7 animals; NDTG: N = 5 animals
